# A comparison of manual and carbon dioxide trap sampling of *Ornithodoros* soft ticks from warthog resting sites in South Africa

**DOI:** 10.1186/s13071-025-06896-8

**Published:** 2025-07-28

**Authors:** Cynthia Mapendere, Armanda D. S. Bastos, Etter Eric, Livio Heath, Ferran Jori

**Affiliations:** 1 Department of Zoology and Entomology, University of Pretoria, Pretoria, South Africa; 2 Hans Hoheisen Research Centre, Department of Veterinary Tropical Diseases, University of Pretoria, Pretoria, South Africa; 3 CIRAD, UMR ASTRE, 97170 Petit-Bourg, France; 4 Department of Production Animal Studies, University of Pretoria, Pretoria, South Africa; 5 Transboundary Animal Diseases, Onderstepoort Veterinary Institute, Agricultural Research Council, Pretoria, South Africa; 6 UMR ASTRE, CIRAD-INRAE, Campus International de Baillarguet, 34398 Montpellier, France

**Keywords:** *Phacochoerus africanus*, Argasid ticks, *Ornithodoros moubata* species complex, Disease ecology, Africa, Vectors

## Abstract

**Background:**

In East and Southern Africa, the African swine fever (ASF) virus is maintained in an ancient sylvatic cycle involving warthogs (*Phacochoerus* spp.) and *Ornithodoros* soft ticks inhabiting warthog burrows. Although carbon dioxide (CO_2_) traps have previously been used to collect ticks from pigsties in Portugal, this method has never been tested in the context of the ASF sylvatic cycle in Africa. As warthogs adapt their resting site preferences in response to different levels of habitat transformation, our study aimed to evaluate the effectiveness of CO_2_ traps versus traditional manual collection of soft ticks inhabiting two warthog resting sites: warthog burrows (natural) and house decks (anthropogenic).

**Methods:**

The study was performed in Mjejane Game Reserve, a wildlife conservancy adjacent to the Kruger National Park in South Africa. Sixty-one warthog resting sites (31 natural burrows and 30 house decks) were sampled to compare *Ornithodoros* tick numbers using manual and CO_2_ trap methods during wet (summer) and dry (winter) seasons.

**Results:**

The number of ticks collected with CO_2_ traps (*n* = 2024) was significantly higher than those collected with the manual method (*n* = 885, *P* < 0.001) for both resting site types. Moreover, the number of ticks collected using CO_2_ traps from house decks (*n* = 1399) was significantly higher (*P* < 0.001) compared to burrows (*n* = 625). There were no differences in the number of ticks collected between seasons. Our results suggest that CO_2_ traps are highly efficient for collecting *Ornithodoros* ticks from the two warthog resting site types evaluated in our study area. They also confirm that warthogs can adapt to different levels of habitat transformation and human presence.

**Conclusions:**

The standardised use of the CO_2_ trap method facilitates investigations on the distribution of tick-related ASF cycles in sub-Saharan Africa and improves our understanding of the eco-epidemiology of ASF and other *Ornithodoros* tick-borne diseases.

**Graphical Abstract:**

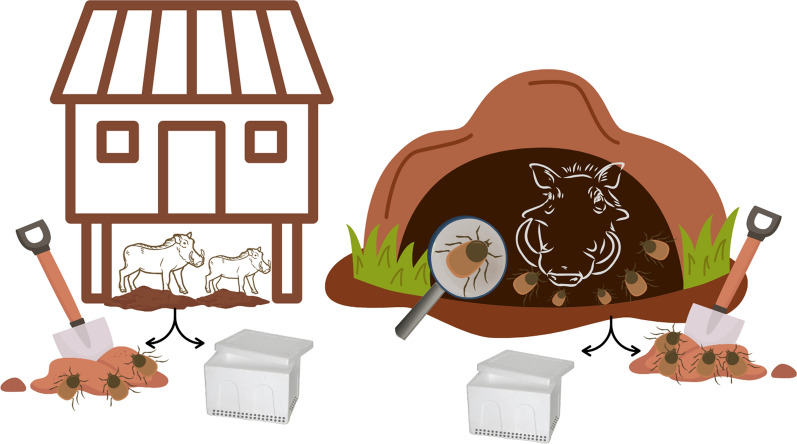

## Background

Vector collection is an integral process in the study of the epidemiology and ecology of vector-borne diseases [1]. Researchers in the field need to have efficient and standardised methods for sampling these vectors, as this allows for comparison of spatial and temporal vector abundance, which is considered a relevant indicator of disease transmission risk [2, 3]. In East and Southern Africa, soft ticks of the *Ornithodoros moubata* species complex inhabit warthog resting sites and are both maintenance hosts and vectors of African swine fever (ASF) virus. As tick-borne transmission of the virus at the wild–domestic pig interface has serious economic consequences for pig production, determining the spatial and temporal distribution of *Ornithodoros* ticks is crucial for developing mitigatory and preventive strategies for the pig sector [4]. This requires the optimisation of tick collection methods.

Diverse techniques have been employed when collecting *Ornithodoros* ticks in different parts of the world. One of these is the manual collection method, which has traditionally been used since the first descriptions of the sylvatic cycle in East and Southern Africa and remains widely used [5–9]. This method involves the use of a shovel to collect soil from subterranean burrows used as overnight refuges by warthogs and passing the soil through a sieve to isolate the ticks [10]. Alternative methods include vacuum aspiration [8, 11] and solid carbon dioxide (CO_2_) traps [12].

Solid CO_2_ (dry ice) traps have been effectively used to collect a broad range of insect vectors, including ticks [13]. The approach centres on the host-seeking behaviour of blood-feeding arthropods in response to host CO_2_ exhalation cues [14]. In addition to being used successfully to collect hard ticks in North America [15], the CO_2_ trap method was also implemented with success for *Ornithodoros* soft tick collection from pigsties in Portugal [12]. Despite this, the method has never been tested for the collection of soft ticks in warthog habitats in Africa. Therefore, the goal of our study was (1) to explore the potential performance of CO_2_ traps in collecting *Ornithodoros* ticks from warthog habitats under field conditions compared with the traditional manual method, and (2) to assess the potential influence of habitat transformation in warthog resting behaviour and tick population loads present in warthog resting sites. The results were analysed and discussed in the context of future studies of the ASF virus sylvatic cycle in sub-Saharan Africa and its potential resilience to ongoing habitat transformation.

## Methods

### Study area

**Fig. 1:**
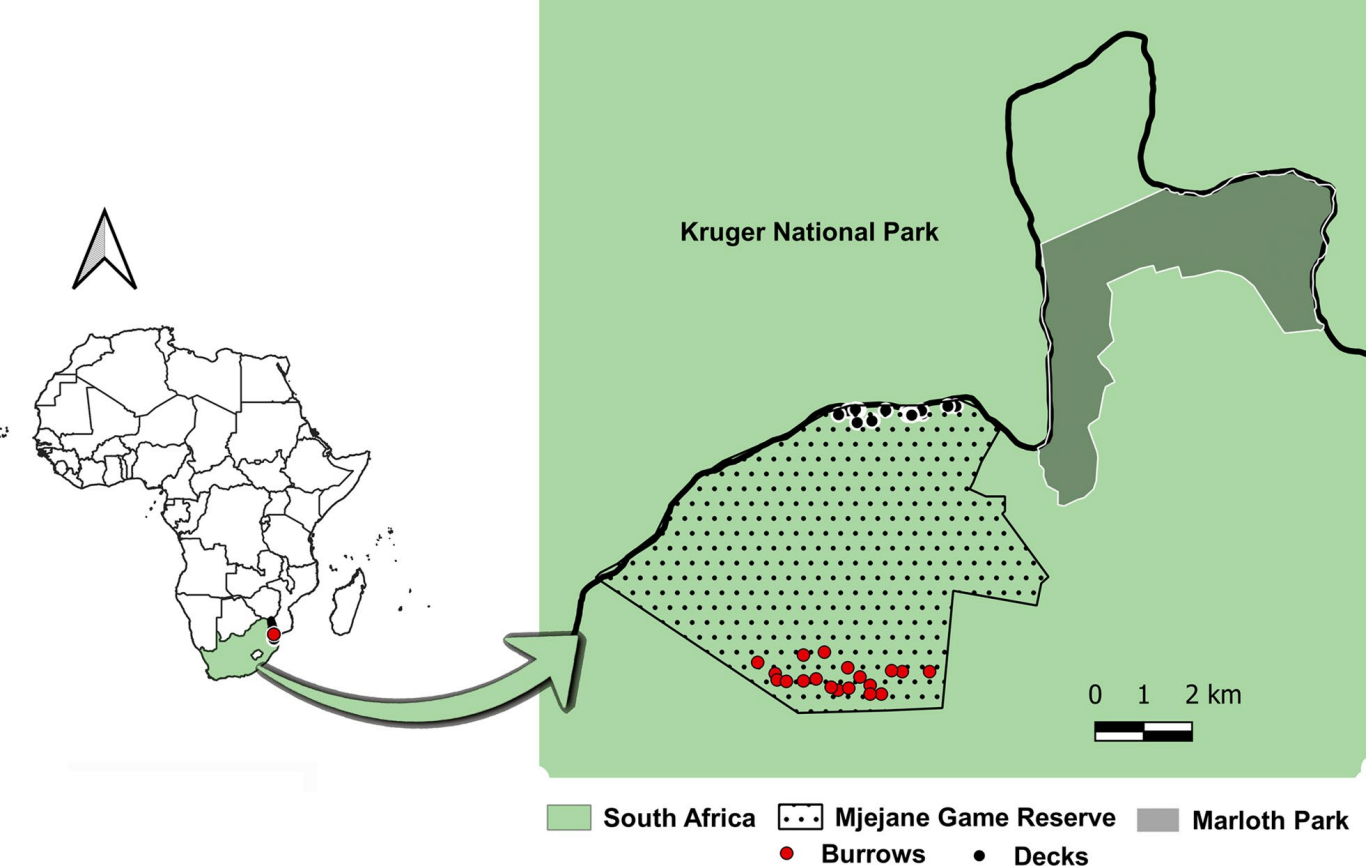
Map indicating the distribution of warthog resting sites (burrows and decks) in Mjejane Game Reserve. The surrounding agricultural and natural areas are also shown

The study was conducted in Mjejane Game Reserve (MGR) (25°20′36″S, 31°46′58″E), which is situated in the Mpumalanga Province of South Africa (Fig. 1). MGR is a private wildlife area measuring 4000 hectares located at the southern boundary of the Kruger National Park (KNP), adjacent to the Crocodile River (Fig. 1). Its eastern and western boundaries are adjacent to large crop farms producing citrus, banana, and sugarcane and human settlements of local communities. There is no physical fence separating KNP and MGR; wildlife species roam freely between both protected areas. The reserve is home to most of the mammals present in the KNP, including warthogs (*Phacochoerus africanus)* and bushpigs (*Potamochoerus larvatus*). The region is characterised by two distinct seasons: the wet/austral summer season (from October to March) and the dry/austral winter season (from April to September). The average annual rainfall in our study area is 638 mm, and the mean annual temperature is 21.9 °C, with wet season temperatures frequently breaching 40 °C.

### Warthog activity in resting sites

The level of activity of warthogs in resting sites was assessed by deploying 20 camera traps (Browning® Trail cameras BTC-8E-HP5) at the entrance of 13 decks and seven burrows for 14 consecutive days in the dry season. The camera traps were set to record 10-s videos each time movement was detected within a 15 m distance from the entrance of the resting site. This video footage was used to determine the presence and number of warthogs entering and leaving a warthog resting site. Data were downloaded from memory cards, and observations (location and metadata) were recorded in Microsoft Excel. The resting site use intensity was calculated by multiplying the number of warthogs using respective resting sites and the time spent within each resting site, quantified as ‘resting hours’. The resting hours were divided by the number of days the camera trap was deployed to provide a standardised index of the use of each resting site expressed as resting hours/day. Standardisation of the sampling effort was required since the duration of camera deployment was not the same across the different resting sites.

### Tick collection and identification methods

Sixty-one warthog resting sites (31 burrows and 30 decks) were identified using field rangers’ knowledge of the study area. Two sampling activities were conducted over 23 days in March (wet season) and 25 days in July (dry season). At each sampling site, *Ornithodoros* ticks were initially collected using the manual method. Once sorted into different life stages and counted, the live ticks were returned to the burrow and the respective sites were sampled 30 min later using the CO_2_ trap method. This was done in order to attempt a direct comparison of the two methods at the same location and with the same available tick population. In order to identify the tick species present, 16S ribosomal RNA (rRNA) gene amplification and sequencing of a subset of samples from decks and burrows was performed, as previously described [16]. The nucleotide sequences were used in BLAST nucleotide searches (www.ncbi.nlm.nih/blastn) against the GenBank database in order to identify the closest sequence matches. A 16S rRNA dataset containing reference sequences for each of the three warthog-burrow-associated *Ornithodoros* species that occur in South Africa [17] and completed with closest matches and Mjejane Nature Reserve (MNR) tick sequences was used to infer a phylogenetic tree.

#### Manual collection

The manual sampling method involved using a 2 m-long shovel to scrape soil from the roof and sides of the warthog burrow and to collect sand from the floor of the deck habitats [8, 20]. The harvested soil was placed in a 5-L bucket and sieved to recover ticks. Two different sieve sizes were used to make it easier to separate the smallest nymphs from fine soil particles. After sieving, the soil was spread onto a black polythene sheet and exposed to the sun. Since ticks are photophobic and move away from the sun, movement could be detected allowing for the collection of all the ticks on the sheet. The soil was also turned several times to allow for exhaustive sampling. Live ticks were returned to the burrow after being sorted into different sizes and counted.

#### Carbon dioxide trap design, calibration, and deployment

**Fig. 2:**
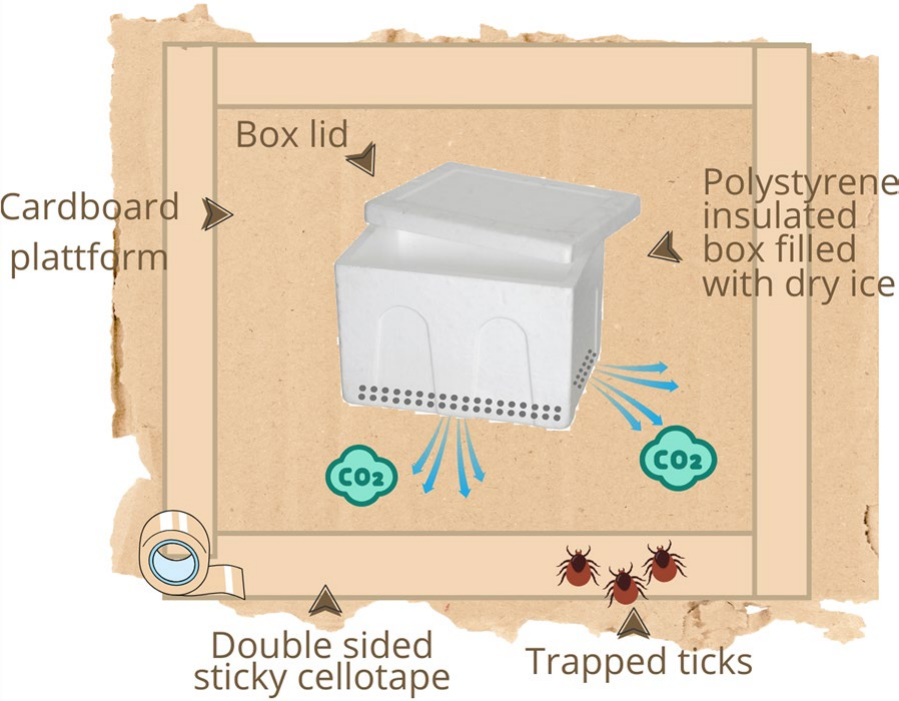
Graphical representation of the carbon dioxide (CO_2_) traps used in the study

The CO_2_ trap (Fig. 2) consisted of a 15 cm × 5 cm × 15 cm Styrofoam box with a volume of 3375 cm^3^. The sides of the box had two rows of c. 2.5 mm diameter holes that were 2 cm apart, and the holes on each row were 2 cm apart. The entire box was fixed to a 25 cm × 25 cm cardboard surface, and a piece of double-sided tape (25 cm long and 1 cm wide) was placed 0.2 cm from each edge of the cardboard surface along each of the four sides of the base.

Prior to field deployment, the CO_2_ traps were calibrated to determine the average time it takes for a pre-determined quantity of dry ice to sublimate into gaseous CO_2_. This was achieved by filling 15 traps with 1000 g of dry ice which were then left in a typical natural warthog habitat until the dry ice had completely sublimated. The average time taken for the dry ice to sublimate was 373.46 ± 33 min. The average temperature in the study area is 25.6 °C during the wet season and 23.2 °C during the dry season [21].

Approximately 30 min after returning ticks collected by the manual method to their respective warthog resting sites, CO_2_ traps were deployed for an average of 360.21 ± 14 min. The ticks captured (stuck on the double-sided tape) were carefully picked using tweezers, placed in labelled, plastic tubes, and transferred to dry ice (−78.5 °C). 

### Sampling effort

Due to the differences in tick collection methods, the calculation of the sampling effort was divided into three parts: preparation time, hands-on time, and hands-off time. Preparation time is the time it took to assemble the tick collection tools and materials for each sampling process. Hands-on time for the CO_2_ method refers to the total time invested between the deployment of the traps and collection of the last trapped tick minus the time the traps were in the respective resting sites (hands-off time). The hands-on time for the manual method refers to the period invested from the point of sand collection in warthog habitats up until the picking and storage of the ticks.

### Tick preservation and classification

Ticks were kept on dry ice during transport to the Onderstepoort Veterinary Institute laboratory, where they were stored at −80 °C until further processing. At the laboratory, ticks were sorted according to size using sieves with different mesh sizes which correspond to different developmental stages [6], allowing for rapid sorting of ticks into adult and nymphal stages.

### Statistical analysis

Statistical analyses were carried out using R version 4.3.0 (R Core Team, 2021). The data were tested for normality using the Shapiro–Wilk test. As data were not normally distributed, the Wilcoxon signed-rank test was used to test for differences of means among groups. A Wilcoxon signed-rank test with continuity correction was conducted to evaluate whether CO_2_ traps were more effective in capturing ticks from warthog resting sites than the manual method. The data were tested for differences due to the type of resting site (decks or burrows), season (dry or wet), and life or developmental stages (adults and nymphs) of the ticks collected. A Spearman’s correlation test was conducted to determine the nature of the relationship between the number of ticks collected using the CO_2_ method and site-use intensity measured using the metric of resting hours. The predictor variable used was warthog resting hours/day, and the response variable was number of ticks. A Mann–Whitney *U*-test was used to calculate the influence of seasonality on tick abundance in resting sites.

## Results

### Molecular confirmation of *Ornithodoros* species in Mjejane Nature Reserve

Sequencing of the 16S rRNA gene of 16 ticks confirmed that all were identical across the gene region characterised. The 16S rRNA phylogeny further confirmed that the species present, both in burrows and in decks, was *Ornithodoros phacochoerus*. Sequences generated in this study are available under Genbank accession numbers PV943752-PV943767.

### Warthog activity levels in resting sites

A total of 1262 videos corresponding to 238 camera trap days were obtained and analysed. During the observation period, 12 out of the 20 cameras (60%) detected some level of warthog activity in seven burrows and 13 house decks (Table 1). All monitored resting sites were used continuously for an average of 3.16 ± 2.69 days, and the longest period recorded at a given site was 10 days (Table 1). The average number of activity days per resting site type was not significantly different between decks and burrows. Although the total number of warthog individuals was 3.6 times higher in decks than in burrows, the average number of warthogs using each habitat type was not significantly different, i.e. 2.20 ± 1.05 individuals in burrows versus 2.27 ± 0.47 in decks (Table 2). Other non-target species that were captured on camera traps walking past house decks included impala (*Aepyceros melampus*), nyala (*Tragelaphus angasii*), and bushbuck (*Tragelaphus sylvaticus*) antelope.

**Table 1:**
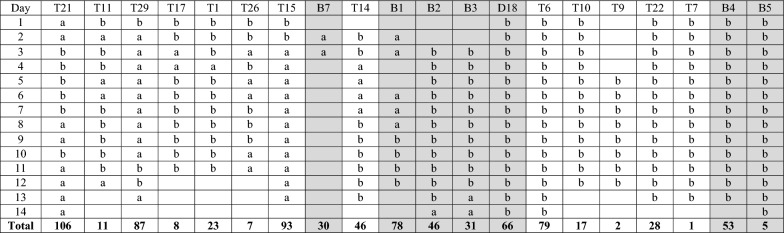
Frequency of resting site use by warthogs captured after 14 days of camera trap observation in burrows (in grey) and house decks (in white)

The absence of values refers to the lack camera trap deployment. Totals in bold refer to the number of ticks collected in every resting site

^a^Resting site used by warthogs

^b^Resting site not used by warthogs

**Table 2:** Comparative indicators of warthog activity in different types of resting sites (burrows versus house decks)

	Number of individuals	Number of warthogs/days	Max	Min	Number of ticks
Deck	36	2.3	5	1	2024
Burrow	10	2.2	5	0	871

### Relationship between warthog activity and tick abundance

The number of resting sites with warthog activity and tick presence was limited (*n* = 12), encompassing nine decks but only three burrows. However, mean tick burdens were not significantly different (median 52, interquartile range [IQR] 31–78) in burrows versus decks (median 30 [IQR 11–87]). There was a significant positive correlation between resting site activity measured in resting hours/day (Table 2) and tick abundance (*rs* = 0.503, *P* = 0.024). The Spearman coefficient of correlation suggested a moderately strong relationship between both variables. However, the small number of observations in burrows (*n* = 3) was insufficient to draw reliable conclusions about this association.

### Comparison of the efficacy and effort of the two tick collection methods

**Fig. 3:**
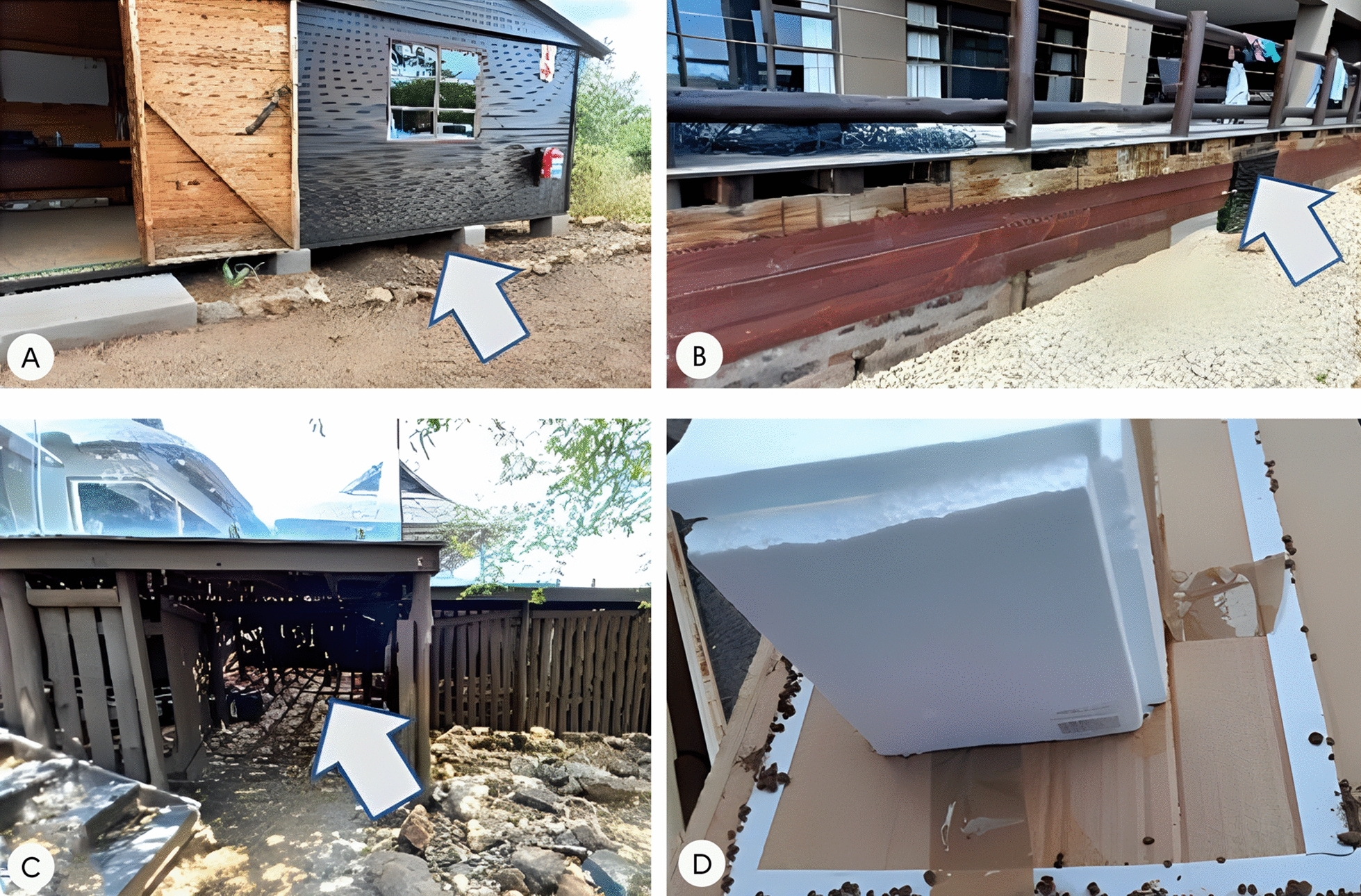
Pictures showing **A**–**C** some of the decks occupied by warthogs (the arrows show the places where warthogs rest) and **D** ticks captured on the adhesive tape around the polystyrene box releasing CO_2_

Carbon dioxide traps (Fig. 3) captured 2024 ticks (per site mean [*M*] = 33.18, standard deviation [SD] = 39.26), while only 885 ticks (per site *M* = 14.50, SD = 19.12) were captured using the manual method (Table 3). A Wilcoxon signed-rank test conducted revealed that the number of ticks collected using the two methods was significantly different, *Z* = −4.63, *P* < 0.001. Ticks were collected from 99% of the sites sampled using the CO_2_ trap approach, and the highest number of ticks from a single site was 187 specimens. When using the manual method, ticks were collected from 50 of the 61 resting sites sampled, representing an 82% infestation rate. The highest number of ticks collected from a single resting site using the manual method was 97.

**Table 3:** Tick developmental stages in different seasons

Developmental Stage	CO_2_ trap method				Manual method			
	Season (wet)	Season (dry)	Habitat type (burrow)	Habitat type (deck)	Season (wet)	Season (dry)	Habitat type (burrow)	Habitat type (deck)
Nymphal stage 1	35	83	64	54	16	35	25	26
Nymphal stage 2	59	255	112	202	26	65	21	70
Nymphal stage 3	312	420	154	578	145	146	73	218
Nymphal stage 4	125	121	99	147	82	50	69	63
Nymphal stage 5	471	105	178	398	220	84	108	196
Adult	6	32	18	20	3	13	8	8
Total	1008	1016	625	1399	492	393	304	581

Considering sampling effort, CO_2_ traps required a shorter hands-on time per site (16 ± 3 min) compared to the manual method which required 44 0.03 ± 0.68 min per site. On average, it took the researcher and a single helper 10.53 ± 0.23 min for soil collection, 27.59 ± 0.6 min for tick detection and collection from the soil, and 5.87 ± 0.19 min to count collected ticks while using the manual method. The average hands-off time for the CO_2_ method was 360.21 ± 14 min. Finally, CO_2_ traps had a longer preparation time, requiring 11 ± 1.6 min to make each trap.

### Comparison of tick abundance in different warthog resting sites

With the use of CO_2_ traps, a total of 625 ticks were collected from burrows (*M* = 19.53, SD = 35.64), while a total of 1399 ticks (*M* = 48.24, SD = 38.06) were collected from decks (Table 3). A Wilcoxon test revealed that this difference was significant, *Z* = 5.83, *P* < 0.001.

### Tick developmental stages according to sampling method

Regardless of the tick collection method used, nymphs were the most abundant tick stage collected, with N1 and N3 having the highest number (Table 3). The Chi-squared test of independence revealed a significant association between the method of collection and the distribution of tick developmental stages, *χ*^2^ = 45.28, *df* = 6, *P* < 0.001. The observed distribution of developmental stages differed significantly between the CO_2_ trap and manual methods (Table 3). The number of nymphs collected was consistently and significantly higher with the CO_2_ trap method.

### Influence of seasonality on tick abundance in resting sites

During the dry season, 1016 (*M* = 26.05, *SD* = 19.52) ticks were collected using the CO_2_ method, while 1008 (*M* = 45.81, *SD* = 58.76) ticks were collected during the wet season using the same method (Table 3). A Mann–Whitney *U*-test revealed that there were no statistically significant differences between the number of ticks collected in wet and dry season, *U*_(122)_ = 1714, *Z* = −0.11, *P* = 0.99, indicating that the distributions of the number of ticks collected in different seasons are similar.

## Discussion

In East and Southern Africa, *Ornithodoros* soft tick presence in warthog burrows is well documented. Their occurrence is very specific and does not require specific identification methods at the genus level. Tick collection for the investigation of their presence as indicators of a potential occurrence of the ASF sylvatic cycle [22–24] have been traditionally implemented manually. This study is the first attempt to test the efficacy of using CO_2_ traps (solid CO_2_) to collect *Ornithodoros* spp. ticks from natural and built warthog resting sites. The study demonstrates that CO_2_ traps are more effective than the traditionally described manual method for capturing *Ornithodoros* ticks from warthog habitats.

A key advantage of using CO_2_ traps is that more ticks are collected per unit time than with the manual method. Considering that various studies in East and Southern Africa suggest that the prevalence of African swine fever virus (ASFV) in *O. moubata* ticks is often very low [20], a method that increases the researchers’ throughput, such as the CO_2_ trap method, will be a good alternative since it makes it possible to collect of larger numbers of ticks samples, increasing the odds of detecting the ASF virus.

Another advantage of using CO_2_ traps is that they can be deployed in several warthog resting sites simultaneously. This improves the standardisation of the sampling protocol since important factors, such as the time of day of trap deployment and sampling effort, will be much easier to keep constant. This is important in the context of tick sampling since tick activity differs depending on the time of day [25]. Furthermore, the ability to deploy multiple traps simultaneously across different habitats or geographical locations increases the spatial coverage and representativeness of the sampling effort.

Also, despite the lengthy hands-off period (360.21 ± 14 min), the traps require minimal human involvement, making them more efficient in a time-constrained setting. While the manual method is cost-effective (purchasing of equipment is a once-off cost), one of its limitations is that it is labour-intensive and requires a more hands-on or ‘active’ approach. Also, its success is limited to how far a shovel can reach into the warthog resting site. The CO_2_ traps, on the other hand, can lure ticks that are at the periphery of warthog resting sites. This allows the possibility to standardise the sampling effort, thereby improving reliability and comparability of data from different sources or areas.

Though the discussion has suggested that CO_2_ traps are more effective in tick collection, this approach has a major limitation in that it requires the compulsory availability of dry ice. This can be a significant constraint when sampling in remote areas, distant from readily available dry ice sources. In addition, purchasing dry ice has higher cost implications, and depending on the number of warthog habitats that need to be investigated, its long-term preservation in field conditions can be challenging. Though this can be mitigated by using a portable dry ice maker, the portable dry ice maker and the compressed liquid CO_2_ needed to make the dry ice are expensive.

The study also revealed the existence of a correlation between warthog activity and the occurrence and/or abundance of ticks in warthog resting sites, which was higher in artificial than in natural warthog resting sites. This resting site preference was associated with a higher tick population number in decks compared to burrows. This is consistent with previous observations of a correlation between tick abundance and host availability reported by O’Neill and White [26] in the context of a generic mathematical model on tick–host associations. In that case, the higher abundance of warthogs in house decks is possibly related to the larger spaces provided by these structures compared to burrows and potentially better protection against predators. However, our number of monitored burrows was not very large (*n* = 3), and these results should be confirmed with a larger number of monitored resting sites.

In addition to the influence of anthropogenic structures (decks) on tick abundance, the number of ticks could also be potentially affected by season [12, 17]. In other studies, such as Nevill [14] and Bouchard and Dibernardo [27], it was observed that tick population numbers could increase during certain seasons of the year (i.e. hot and wet compared to the cold seasons). This hot and wet season equally coincides with the warthog breeding period. During this time, there is likely to be cooperative breeding [19], which implies more frequent burrow use by farrowing females and therefore more host availability for the ticks. This could positively influence tick abundance and is supported by the positive relationship between this parameter and the level of warthog activity observed in our study. However, our study was not designed to determine the role of season on tick abundance.

An additional observation in our study was the higher number of nymphs compared to adult stages that were collected regardless of the method used. This finding is consistent with other studies conducted in Southern Africa [5, 11, 22, 23, 28], which also found more nymphs than adults in warthog burrows. A possible explanation could be that because of their size, nymphs are likely to be more mobile than adults. Ginsberg and Ewing [29], while using CO_2_ traps to sample hard ticks, found that individuals with higher mobility tend to be captured at a higher rate than those that move at a slower pace. Furthermore, as with any organism, some will die before reaching adulthood, and this intrinsic mortality rate [30] means one is more likely to capture more nymphs compared to adults. Given this potential bias towards life stages with greater mobility, CO_2_ traps can be recommended for studies that are not concerned with the life stages of sampled ticks. The CO_2_ traps are also useful when targeting nymphs because, due to their small size, nymphs are more likely to be missed if sampled using the manual method.

One of the limitations of our findings is the limited observation time and the reduced distribution of our sample in the study area, which makes it difficult to raise more robust conclusions on the generalisation of our results. Moreover, other ecological factors that may have influenced host abundance, such as proximity to food and water [18], could not be measured in this study.

Though the researchers concluded that CO_2_ traps were superior if compared with the manual approach, some limitations can impact the credibility of the study. It can be presented that the differences between the two methods could have been biased by the sampling protocol. The sampling protocol involved collecting the ticks using the manual method first, releasing them back to the resting site, and then re-sampling them with the CO_2_ traps. This could have potentially increased the number of ticks attracted by CO_2_ traps. Unfortunately, the reverse treatment (CO_2_ trap followed by manual) was not possible because removing ticks from the double-sided tape is more likely to harm the ticks making the harmed ticks impossible to re-sample. Nevertheless, the conclusion that CO_2_ traps are more effective can be further supported by the observation that the differences in the number of ticks collected using the two methods were highly and consistently significant. Moreover, at some locations (*n* = 3), ticks were only recovered using the CO_2_ method, strengthening the conclusion that the CO_2_ trap method is more efficient compared to the manual collection method.

This study can be considered as a first step to standardise the collection of *Ornithodoros* ticks in Africa, an important aspect in studies concerned with furthering our understanding of ASF ecology within the sylvatic cycle. The study clearly documents how CO_2_ traps can be used in the sampling of *O. moubata* ticks in both natural burrows and anthropogenic structures such as houses with raised floors or decks. It also shows the differences in efficacies of the CO_2_ and manual methods. This is important given that in many African countries, the presence or distribution of the ASF sylvatic cycle has not been extensively investigated [20]. From that perspective, the availability of a more efficient method can facilitate this process.

Our work focused on ticks in warthog resting sites due to the importance of this kind of environment in the study of the sylvatic cycle of ASF. However, our results for tick collection in decks provide evidence that the method is efficient in surfaces as large as 25 m^2^. This suggests that the method holds potential for collecting *Ornithodoros* ticks in larger areas, such as domestic pig premises. The domestic cycle between pigs and *Ornithodoros* ticks has been seldom demonstrated in sub-Saharan Africa. This is despite the fact that this cycle represents a suspected cause of increased resistance to the pathogenic effects of ASFV observed in some populations of domestic pigs regularly exposed to the virus [31, 32].

Also, according to our knowledge, this is the first study to document warthog adaptation to artificial resting sites and infestation of these sites with *Ornithodoros* ticks. These types of warthogs resting sites are likely to expand with the development of eco-tourism in Africa. While this expansion might not have a major impact on the maintenance of the sylvatic cycle, it does facilitate the proximity of soft ticks to human habitations increasing the exposure of humans and their livestock to *Ornithodoros* tick bites. This suggests that warthog populations are quite resilient to structural transformations but that the sylvatic cycle can readily become established in more anthropised environments, with potential implications for disease transmission to animals and humans. This is relevant as *Ornithodoros* ticks such as *O. sonrai* have been found to vector *Borrelia crocidurae* in East and West Africa, the causative agent of relapsing fever in humans [33].

## Conclusions

In conclusion, our results provide evidence that CO_2_ traps represent a very efficient tool to improve the collection of *Ornithodoros* ticks from warthog habitats, compared to traditional manual methods. Its application could facilitate investigations to improve our understanding of the role, presence, and distribution of *Ornithodoros* ticks in the sylvatic cycle and potentially in domestic pig–tick cycles of ASF in sub-Saharan Africa. In addition, our study highlights the common warthog’s capacity to adapt to environmental changes such as the expansion of human habitation structures into natural habitats, generating new opportunities of interactions between *Ornithodoros* ticks’ populations, humans, and their domestic animals. Considering the strong and fast drivers of change likely to occur in Africa in the next decades, the capacity of the ASF sylvatic cycle to adapt and evolve with those changes deserves further investigation. Therefore, with careful logistical planning, the CO_2_ method can be a very effective tool for soft tick collection, which could help facilitate investigations on the ecology of ASF.

## Data Availability

Data supporting the main conclusions of this study are included in the manuscript.

## Abbreviations

ASF: African swine fever
ASFV: African swine fever virus
KNP: Kruger National Park
MGR: Mjejane Game Reserve
SD: Standard deviation
